# Investigating Information Visualization to Combat Information Overload in Electronic Health Records: Protocol for a Randomized Controlled Trial

**DOI:** 10.2196/74247

**Published:** 2025-09-09

**Authors:** Saif Khairat, Jennifer Morelli, Marcella H Boynton, Thomas Bice, Jeffrey A Gold, Shannon S Carson

**Affiliations:** 1 Carolina Health Informatics Program University of North Carolina at Chapel Hill Chapel Hill, NC United States; 2 School of Nursing University of North Carolina at Chapel Hill Chapel Hill, NC United States; 3 Sheps Center for Health Services Research Chapel Hill, NC United States; 4 School of Medicine University of North Carolina at Chapel Hill Chapel Hill, NC United States; 5 School of Medicine Oregon Health & Science University Portland, OR United States

**Keywords:** electronic health records, information visualization, cognitive fatigue, information overload, eye-tracking, EHR usability

## Abstract

**Background:**

Electronic health records (EHRs) have been linked to information overload, which can lead to cognitive fatigue, a precursor to burnout. This can cause health care providers to miss critical information and make clinical errors, leading to delays in care delivery. This challenge is particularly pronounced in medical intensive care units (ICUs), where patients are critically ill and their EHRs contain extensive and complex data.

**Objective:**

We propose to study the effect of an information visualization dashboard on ICU providers’ cognitive fatigue in 4 major US medical centers. In this randomized controlled trial, we will compare 2 leading EHRs with a visualization dashboard–based EHR called AWARE.

**Methods:**

This crossover randomized trial will collect physiological and objective data using a screen-mounted eye-tracking device to assess cognitive fatigue among ICU providers. The study will involve 120 ICU providers from 4 US medical centers, with each site using its institutional EHR as the control and the AWARE EHR as the intervention. Participants will be randomly assigned to use either their institutional EHR or the AWARE EHR first, followed by the other EHR, with the order of EHR use and patient case order randomized by the study team. The AWARE tool is designed to integrate within an existing EHR system and provides clinical support tools, such as the ability to trend data points from body systems at a glance. Data analysis will include eye-tracking metrics, performance measures, and validated surveys to evaluate EHR usability and its impact on clinical decision-making. Primary outcomes include the number of cognitive fatigue instances per patient case, time to complete each case, overall usability of the session, number of mouse clicks per case, provider performance scores from questions asked during each patient case, and perceived usability of each EHR. Secondary outcomes include the number of eye fixations per patient case and perceived workload of each EHR.

**Results:**

This EHR usability study was funded in 2021 and initiated in 2022, with a completion date of 2025. Data collection began in August 2023. As of now, 3 of the 4 study sites have completed data collection, with a total of 113 completed sessions thus far. Data collection is ongoing at the fourth site. Preliminary analysis is ongoing, and we expect to begin publishing results in late 2025.

**Conclusions:**

Findings from this research may inform improvements in EHR interface design and usability, which may enhance provider performance, streamline care delivery, and improve patient safety outcomes.

**Trial Registration:**

ClinicalTrials.gov NCT05937646; https://clinicaltrials.gov/study/NCT05937646

**International Registered Report Identifier (IRRID):**

DERR1-10.2196/74247

## Introduction

Providers experience information overload when using electronic health records (EHRs) [1-3]. Providers report that information overload is among the major challenges to EHR usability [1]. Information overload can contribute to cognitive fatigue, causing increased mental effort and difficulty making decisions [4].

Data characteristics, such as quantity, frequency, complexity, and quality, can contribute to information overload [5]. Also, difficulty locating information in the EHR or distinguishing erroneous information can contribute to information overload [6]. EHR systems, while beneficial, often contribute to information overload due to their design and usability issues, leading to cognitive overload and potential errors [7,8].

Information overload is associated with provider frustration, as they reportedly spend up to half of a workday working on EHRs and an additional 1-2 hours at home [9]. EHRs containing too much irrelevant clinical information can hinder understanding and make it challenging for providers to quickly access necessary data [1]. This may lead to potential errors in clinical decision-making.

EHR interface design can contribute to information overload by presenting excessive or fragmented data that overwhelms clinicians. Many EHRs require providers to navigate multiple screens to gather relevant patient information, leading to inefficiencies and increased cognitive burden [10]. Additionally, these systems often display large amounts of data without clear prioritization, making it difficult to quickly extract critical insights [11]. Poor usability and complex navigation further exacerbate the issue, forcing clinicians to spend more time searching for information rather than making informed decisions [12]. Improving EHR design by prioritizing essential information and enhancing data representation is critical to reducing cognitive overload and improving patient care.

Prior literature has suggested that integrating data visualization can help mitigate the negative effects of information overload in EHRs [1,13,14]. However, the mechanisms for evaluating the impact of data visualization on EHR information overload are unknown. Data visualization integrations in EHRs can be defined as tools or systems that aggregate patient health data and visualize it to highlight clinically meaningful patterns. These tools may assist clinicians in information prioritization and in recognizing trends in health data.

Visualization dashboards represent a promising approach to reimagining how data is presented within EHRs [15]. By integrating advanced data visualization techniques, these dashboards can transform large volumes of complex data into easily interpretable formats, allowing providers to quickly grasp essential information [16]. The ability to prioritize and highlight critical clinical data not only reduces cognitive load but also enables clinicians to focus on decision-making rather than becoming mired in assessing irrelevant or fragmented information [17].

Visualization dashboards offer a practical way to improve how data are presented in EHRs [14]. Visualization techniques can simplify the presentation of complex information, enabling providers to swiftly identify essential details without becoming overwhelmed [15]. Rather than navigating through multiple screens and fragmented data sources, providers can access the most pertinent clinical information at a glance [18]. This approach not only alleviates the cognitive burden but also allows providers to concentrate more on patient care.

A potential benefit of visualization dashboards is their ability to streamline workflow [14]. Traditional EHRs often require excessive clicking and searching to find relevant details, whereas a well-designed dashboard may consolidate essential patient data into a single, intuitive view [19]. This may not only save time but also enhance efficiency, giving providers more opportunities to engage with patients and make timely decisions [15].

Furthermore, visualization dashboards can facilitate better data interpretation using interactive elements. For instance, dynamic graphs and charts that allow users to manipulate data presentations can enable providers to explore various dimensions of patient information [20]. This interactivity engages users and enhances their understanding of patient conditions, hypothetically leading to better clinical outcomes [21].

Additionally, eye-tracking technology offers an innovative method for quantifying cognitive load and usability [22]. Measuring pupillometry metrics such as pupil dilation provides objective insights into the cognitive demands of intensive care unit (ICU) providers when interacting with their institutional EHR interfaces versus a visualization dashboard [23]. Such empirical data are essential for assessing both interfaces and identifying design elements that reduce information overload and improve user experience, ultimately informing evidence-based EHR enhancements [20,24].

To determine the extent to which EHRs contribute to cognitive load, prior research has used tools such as usability questionnaires [25]. An innovative approach to measuring cognitive load is the use of eye-tracking technology, which provides objective measures of eye data including measures of pupil size, eye fixations or movements, and eye openness [26]. These measures are associated with cognitive load, information processing, and cognitive fatigue.

This study protocol highlights our approach to examining the impact of information visualization on EHR information overload and usability outcomes. In this study, we will use objective measures such as eye-tracking along with subjective measures of usability to determine where and when information overload occurs when using EHRs. We hypothesize that a visualization dashboard such as AWARE [27] will result in lower cognitive fatigue and higher usability scores compared to institutional EHRs. Additionally, we hypothesize that the order of exposure to the EHR systems will not significantly influence the primary outcomes, as the randomization process will control for any potential order effects.

## Methods

### Overview

We will conduct a multisite, cross-sectional usability assessment of cognitive fatigue and EHR usability in a crossover randomized controlled trial (RCT). The decision to use a crossover RCT design for this study is based on several key considerations. A crossover design allows each participant to serve as their own control, which enhances the statistical power of the study by reducing the variability associated with individual differences. This is especially important in usability studies, where subjective experiences and cognitive fatigue levels can differ significantly among participants. This RCT is registered with ClinicalTrials.gov under ID NCT05937646. This study is a continuation of our prior usability study, and additional information is available in our previously published protocol [28].

This study compares 2 leading EHRs with a visualization dashboard–based EHR, AWARE. AWARE is designed to integrate within an existing EHR system. For the purposes of this study, AWARE is used as a standalone EHR tool. AWARE provides clinical support tools, such as the ability to trend data points from body systems at a glance.

Study participants will be medical ICU providers in 4 US medical centers. Across the 4 participating sites, 2 leading EHRs are used. Each site’s institutional EHR will be used as the control, and the AWARE EHR will be used as the intervention. Although the ICUs at these medical centers differ in terms of catchment area and patient population, all 4 medical centers are designated as level 1 trauma centers and are equipped with level 3 ICU capabilities. The number of ICU beds across the medical centers ranges from 74 to 200 beds per site.

Providers will be recruited by study teams at each participating medical center site through a combination of email outreach, informational flyers, and word-of-mouth communication. Interested providers will be asked to complete a preliminary screening survey to confirm eligibility to ensure they are current ICU providers or, in the case of residents, that they have completed at least one ICU rotation. Providers who previously participated in the initial round of usability sessions with our study team will be excluded from participation in this study.

Study sessions will be conducted in a simulation laboratory or private space designed to replicate the ergonomic environment of inpatient settings. Standard computer screens from each practice setting will be used with ICU-like ergonomic placement, ambient lighting, and seating.

Prior to each study session, the usability specialist will explain the study purpose and consent forms to providers, emphasizing that the purpose of the study is to evaluate EHR usability rather than assess clinical knowledge. Participants will be informed that their participation is voluntary, and they can decline to participate or withdraw from the study at any time. Following informed consent, each participant will be asked to complete a brief paper-based survey.

**Figure 1:**
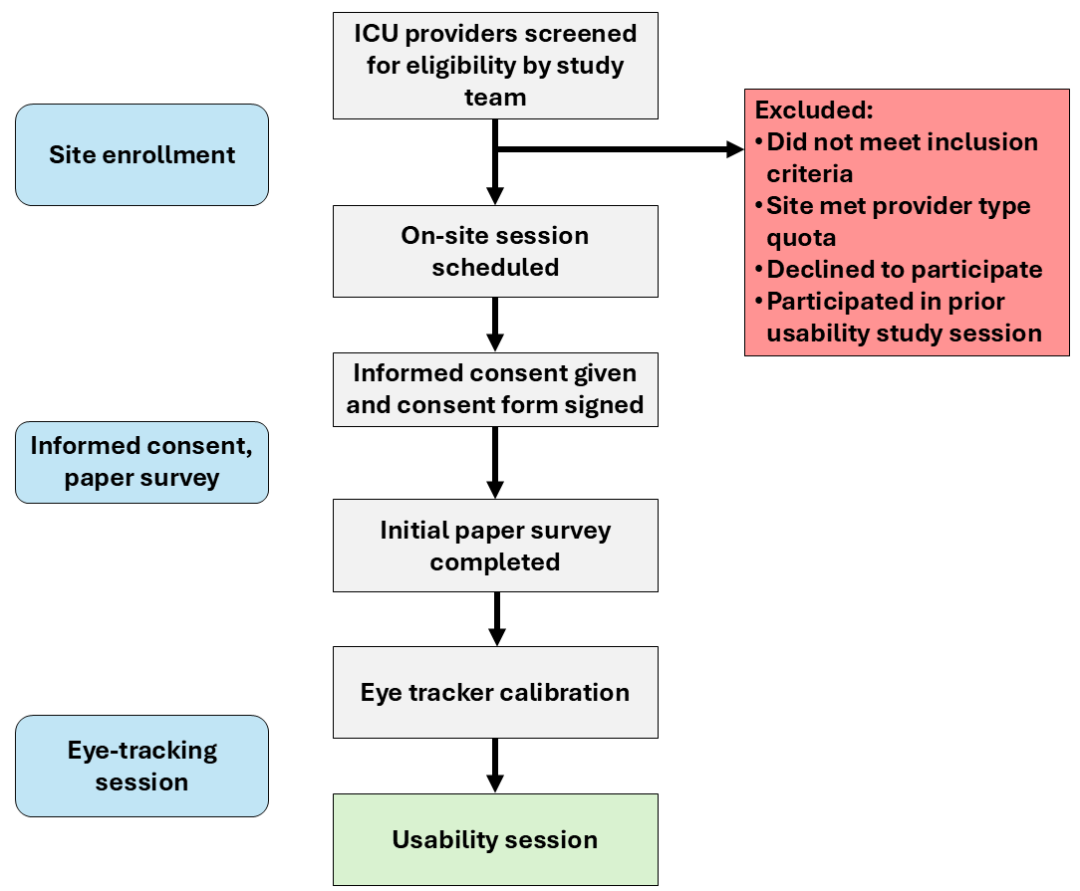
Process map detailing the study steps from site enrollment to the start of the usability session.

We will then ask providers to complete a basic calibration exercise while looking at the monitor, which allows the eye-tracking software to be calibrated to each provider’s eye shape and eye size. We will record pupil diameter and other eye-tracking metrics continuously during the study session. All sessions will use the same screen-based eye tracker. A process map outlining the study procedure from participant enrolment through the eye-tracking calibration is provided in Figure 1.

Providers will be informed that they will use their own institutional EHR for 2 patient cases and the AWARE EHR for 2 patient cases. Both the order of EHR used and patient case order will be randomized by the study team prior to data collection at each site. Providers will be informed that they will review 1 patient case at a time, and that they will begin by performing their usual prerounding tasks on each patient. Providers will then signal to the study team that they are ready to begin the question-and-answer activity for that patient. Providers will be encouraged to continue to use the EHR to answer the questions. The nurse informatician (JM) on the study team will record participants’ answers using both audio and written recordings. After completion of the questions, the provider will then review the next patient record.

The nurse informatician (JM) will provide a brief demonstration of AWARE prior to providers’ use. This demonstration overview includes where to find basic information, such as patient notes, medication information, vital signs, lab results, and how to navigate the patient chart. Providers will be encouraged to ask questions during this demonstration period and are also allowed to ask navigation questions during their patient review. Providers will be instructed that the study team can answer questions related to where to find information or how to display information but that they cannot answer clinical questions related to the patient cases.

### ‎Patient Cases

**Figure 2:**
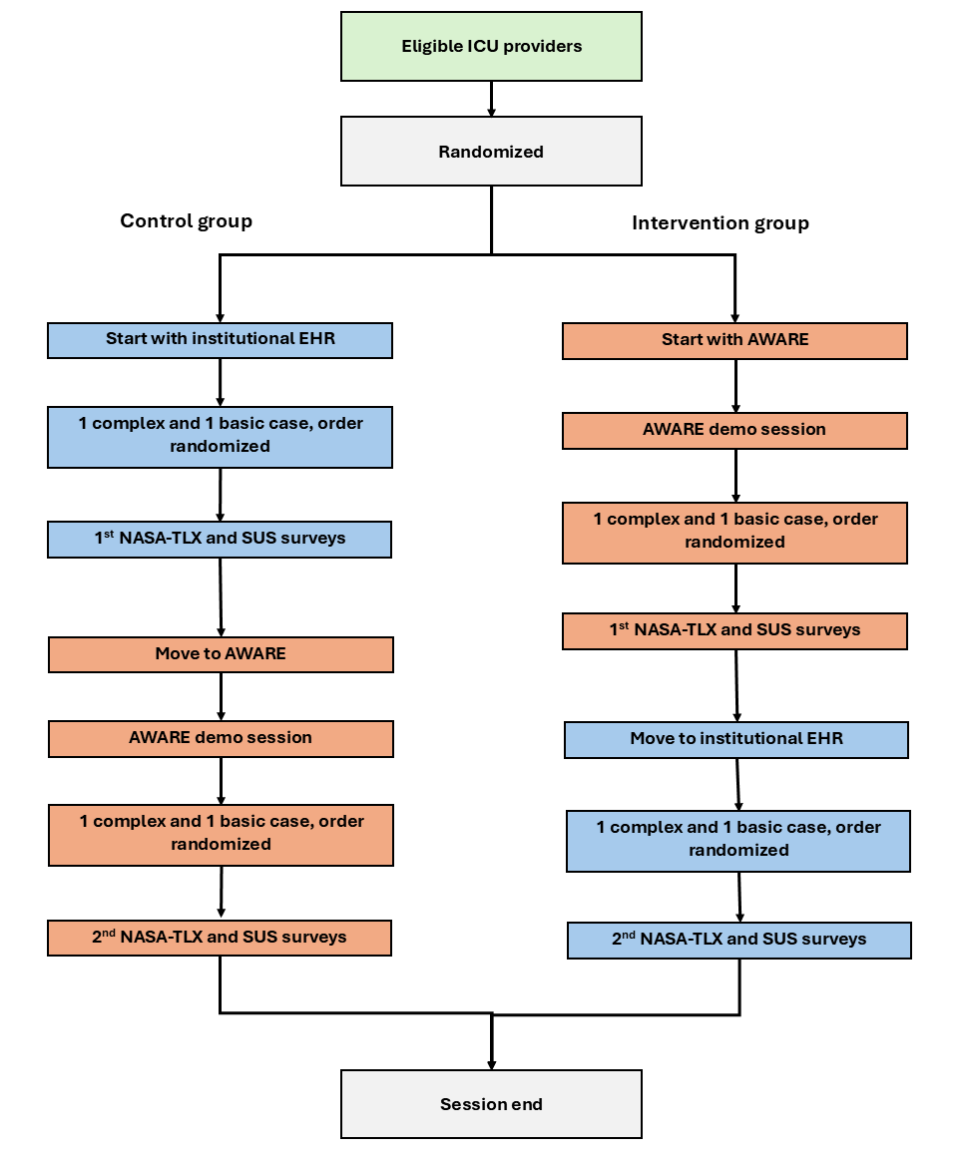
Process map detailing the usability session for participants in both control and intervention groups. EHR: electronic health record; ICU: intensive care unit; NASA-TLX: National Aeronautics and Space Administration task load index; SUS: system usability scale.

The same 4 cases from our prior usability work will be used in this study, which consists of 2 standard ICU-level patients and 2 complex patients. These 4 cases will be used at all study sites, and the nurse informatician (JM) will ensure consistency in patient cases across study sites. Case order will be randomized such that each provider will review 1 standard and 1 complex patient in their institutional EHR and 1 standard and 1 complex patient in the AWARE EHR. The 2 standard ICU-level patients were less critically ill, not currently ventilated, on fewer medications, and had fewer abnormal laboratory values. The 2 complex ICU-level patients were critically ill with many abnormal laboratory values, on a ventilator with poor oxygenation, and on complicated medication regimens. A detailed process map describing the usability session for control and intervention groups can be seen in Figure 2.

### Case Questions

Following a preliminary chart review, providers will be asked 4 questions for each patient case. These questions were developed by a critical care physician (TB) and subsequently reviewed for clarity by a team of critical care physicians prior to data collection. The questions are intentionally designed to ensure that the information could be reasonably located within the EHR and that providers across varying levels of clinical experience would be able to answer. While some questions are relatively straightforward, requiring reference to a single EHR screen or data point, others are more complex, necessitating the integration of multiple data elements or retrieval of information not routinely accessed. This approach allows for analysis of the time required to answer each question, the number of EHR screens accessed, and the number of mouse clicks by each provider. Responses will be evaluated and categorized as correct, partially correct, or incorrect by the nurse informatician (JM) on the research team. Alternative responses will undergo review and scoring by a critical care physician (SSC).

### Sample

Our goal is to recruit 120 ICU providers, 30 at each site, with the following distribution: 20 physicians and 10 advanced practice providers (APPs). The physician group will be divided into 3 subgroups: 5 attending physicians, 5 fellow physicians, 10 resident physicians, and 10 APPs including nurse practitioners and physician assistants. Each study team’s local research assistants will circulate departmental emails and flyers at each site. The site study teams will provide interested individuals with a link to a web-based calendar showing available time slots to facilitate appointment scheduling. Once an appointment is scheduled, the research assistant will email each participant their appointment time and location with a map, as well as a contact number for same-day inquiries or cancellations. Inclusion criteria include the following: ICU physicians and APPs on active full-time ICU service or residents who have completed at least 1 ICU rotation, use of an institutional EHR, and ability to speak English. Providers who participated in our prior usability sessions will be excluded.

### Materials and Software

To measure the extent and effect of cognitive fatigue in the EHR, we will use several tools during the 1-hour sessions.

Providers will be asked to complete 1 paper survey before the eye-tracking session. This survey asks basic demographic questions, including age, gender, years since graduation (from medical school or APP schooling), years of experience with their institution’s EHR, and the estimated number of hours they use their institution’s EHR each week. We will also ask providers if they are on service that day (pre- or postusability session) and to rate their level of sleepiness and stress on a Likert scale.

Directly following the 2 cases in each EHR, providers will be asked to complete 2 additional paper usability surveys. The first usability survey is the National Aeronautics and Space Administration task load index (NASA-TLX) [29], which asks providers to rate the task load of the EHR used for the previous 2 patient cases, including how mentally and physically demanding it was to use and their level of satisfaction and confidence with using the EHR. The second usability survey is the system usability scale [30], which asks providers to rate their satisfaction with the EHR system used, including questions about usability, ease of use, and overall functionality. Both surveys have been used previously among medical providers to measure EHR usability [31,32].

This study will use a noninvasive, screen-based eye-tracker to enhance understanding of providers’ cognitive processing during patient chart review in the EHR. The Tobii Pro Fusion, an advanced eye-tracking device equipped with 2 eye-tracking cameras, captures up to 250 images per second to ensure precise and accurate measurement. The device is designed to mount to the bottom bezel of any monitor, allowing for seamless and unobstructed recording of eye-tracking data. The eye-tracker will be used in conjunction with Tobii Pro Lab software, which enables synchronized recording of both eye-tracking metrics and on-screen activity. Widely used in medical and psychological research, the Tobii eye-tracker and its accompanying software provide detailed data on pupil size, gaze location, and eye movement patterns. These metrics will allow for analysis of information processing behavior, in particular the association between pupil constriction and the study’s outcome variable, cognitive fatigue.

### The AWARE EHR

The 4 patient cases used in the study were built in the AWARE EHR using the same data points as those included in the institutional EHR builds. The team nurse informatician (JM) provided oversight of case builds to ensure consistency and accuracy across platforms.

**Figure 3:**
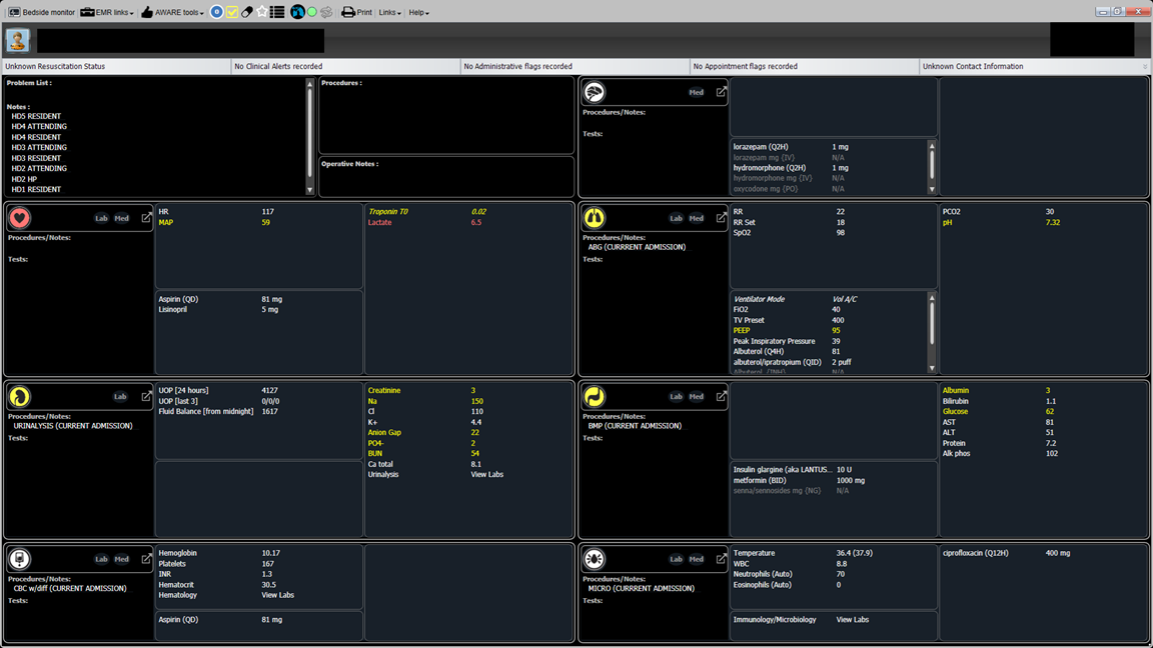
A screenshot of the AWARE patient interface. Each rectangle represents a different system, indicated by that system’s icon (7 in total). Yellow coloring indicates that a value within a system is out of range, while red coloring indicates that a value is significantly out of range.

**Figure 4:**
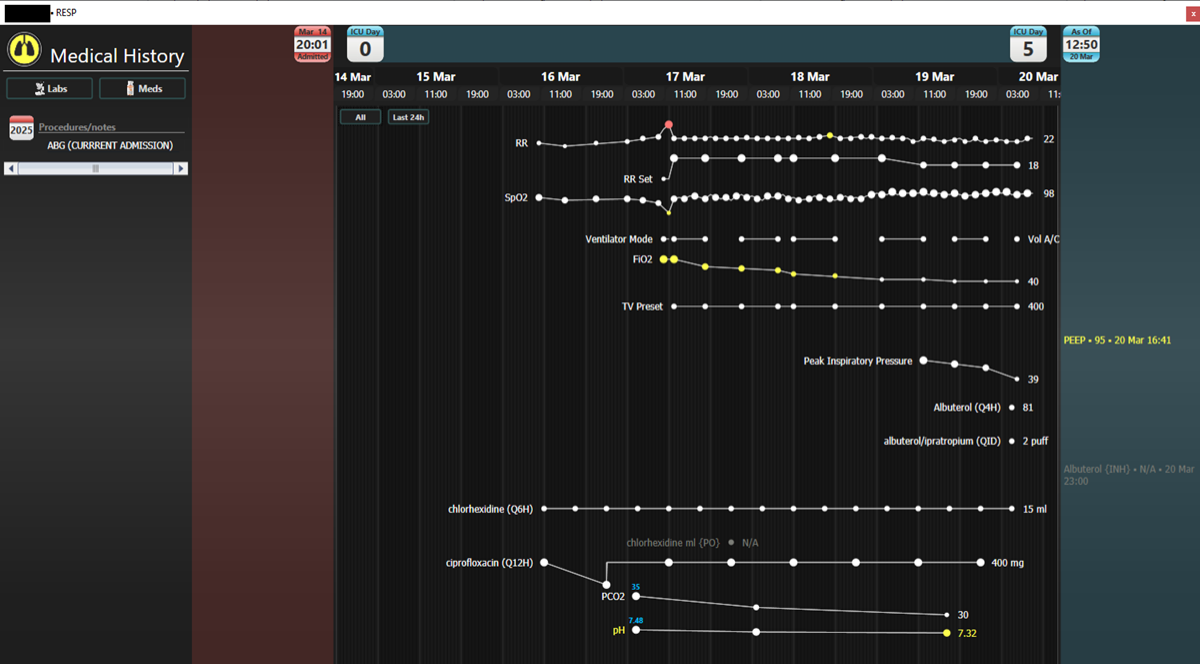
The respiratory system overview within AWARE. Trends are displayed for relevant vital signs and labs, along with medication administration instances along the bottom.

Unlike traditional EHRs, AWARE is structured according to body systems rather than by data type, offering a different approach to clinical information organization. A screenshot of the AWARE EHR is provided in Figures 3 and 4. During the AWARE demonstration, participants will receive instruction on how to access data points for patient charts, including the location for patient notes, medications, vital signs, laboratory values, intake and output, and other clinical data. Participants will be instructed on how to access individual body systems, how to trend individual values, and how to trend data for each system. Participants will be encouraged to ask questions about where to locate data points both during the demo and during the patient case review to ensure access to information needed for completion of the usability session tasks.

### Outcomes

Primary outcomes are cognitive fatigue (using pupil constriction), usability (clicks and completion time), performance score, and perceived satisfaction (system usability scale survey). Secondary outcomes are the perceived EHR workload (NASA-TLX) and information processing (using eye fixations).

To characterize cognitive fatigue, we will use change in pupil size including pupil constriction. We will determine under what conditions providers experience cognitive fatigue while using the EHR. We will determine which patient cases create the highest number of cognitive fatigue instances among providers as indicated by pupil constriction, meaning a rapid decrease in pupil size from baseline. We also seek to understand how cognitive fatigue affects overall EHR usability, the effect of fatigue on performance score, and the perceived satisfaction from providers. Understanding the factors that contribute to cognitive fatigue will fill a significant gap in EHR usability research.

### Analytical Plan

Each participant will receive a single score for each question assigned by a domain expert according to a standardized rubric: 0 for incorrect responses, 0.5 for partially correct responses, and 1 for correct responses. Correct decisions are the aggregate of the correct responses, and errors are the aggregate of the incorrect responses. For each participant, we will compute a score for each patient case and a cumulative score across all 4 patient cases. Case scores will depend on the number of questions asked, such that if a case has 4 questions to be answered, the total score for that case will be equivalent to the highest possible points (4).

Eye-tracking data will provide continuous measurements of pupil size over time for each participant. We will calculate participants’ pupil size based on (1) baseline pupil size at rest, (2) total change in pupil size per case, (3) total change in pupil size per EHR, and (4) total changes in pupil size across the overall session. These measurements will allow for assessment of cognitive load and its variation throughout the different patient cases and task engagements.

We will examine descriptive statistics for both the case and participant characteristics and use multilevel modeling to assess the effect of case-level (eg, complexity) and person-level (eg, gender, professional roles, and site) factors on the outcomes of interest (ie, decision accuracy, usability, fatigue, clicks, time, and number of screens). Exploratory interaction testing between the intervention variable and key case level variables will also be tested. A bottom-up building approach with comparative model fit testing and variable centering will be used. A focus of the models will be the effect of the intervention (AWARE EHR) versus control (institutional EHR). Case variables will be entered into the model as case-level (level 1) predictors. Models will also account for person-level (level 2) factors, such as participants’ gender, age, clinical role, site, and years of EHR experience. We will use SAS 9.4 using PROC MIXED for continuous outcomes and PROC GLIMMIX for binary and count outcomes.

### Ethical Considerations

Recruitment and site testing will occur sequentially such that the study will be implemented at one site at a time, allowing the study team to be present on-site to add organization to the data collection process. Each participant will be required to read and sign a consent form specific to their medical center, witnessed by a representative of that medical center. Participants will be allowed to opt out of the study at any time. We will use 1 screen-based eye-tracker device for data collection at each site. We will recruit providers through flyers and departmental email communications at each site. Participants will be compensated with a $100 gift card for their participation. All data collected will be deidentified and study participants and their data will be assigned a unique identification number. Study data entry and management systems will be secured and password protected to ensure participant privacy and confidentiality. The University of North Carolina at Chapel Hill’s institutional review board approved this study (#20-3384).

## Results

This EHR usability study was funded in 2021. The study was initiated in 2022 with a completion date of 2025. Data collection began in August 2023. Three of the 4 study sites have completed data collection, with a total of 113 completed sessions thus far. Preliminary analysis is ongoing, with publication expected in late 2025.

## Discussion

### Anticipated Findings

This study presents a structured protocol for evaluating the impact of information visualization on mitigating cognitive overload in EHRs. A key strength of this protocol is its multisite, cross-sectional design, which enhances the generalizability of findings by capturing variations in institutional workflows and patient populations. By incorporating an RCT framework, the study minimizes bias and strengthens the validity of its conclusions, providing a robust foundation for understanding how EHR design influences clinician workload and decision-making.

The anticipated main findings of this study are that the AWARE will result in lower cognitive fatigue and higher usability scores compared to the institutional EHRs. Additionally, we hypothesize that the order of exposure to the EHR systems will not significantly influence the primary outcomes, as the randomization process will control for any potential order effects.

### Comparison to Prior Work

Prior literature has suggested that integrating data visualization can help mitigate the negative effects of information overload in EHRs [1,13,14]. Visualization dashboards represent a promising approach to reimagining how data are presented within EHRs [15]. By integrating advanced data visualization techniques, these dashboards can transform large volumes of complex data into easily interpretable formats, allowing providers to quickly grasp essential information [18]. This study builds on previous research by using a visualization dashboard, AWARE, and assessing its impact on cognitive fatigue and usability outcomes in a real-world clinical setting [14].

### Strengths and Limitations

A central focus of this protocol is real-world applicability. By creating near-real-world ICU patient data and simulating inpatient environments, the study ensures that findings reflect actual clinical workflows rather than controlled laboratory settings. This approach facilitates a nuanced assessment of how information visualization tools, such as AWARE, can be integrated into existing systems without disrupting provider efficiency. Maintaining ecological validity is critical to ensuring that usability improvements translate into meaningful enhancements in clinical practice.

Finally, this study will be conducted in multiple medical centers that use the 2 most prominent EHR systems in the United States to ensure the generalizability of the findings. By integrating methodological rigor with real-world relevance, this protocol contributes to the ongoing effort to optimize EHR usability, reduce clinician burden, and enhance patient care.

While we propose to recruit 120 ICU providers, we understand that recruiting this exact number of participants may be challenging. To help mitigate this challenge, we expanded recruitment to include medical ICUs in other affiliated hospitals within the same health care system. All study sites include multiple hospitals to enable recruitment within the same health care system under the same institutional review board.

Variations in EHR interface design and performance may affect study findings. To help mitigate this effect, we selected study sites that use the 2 most prominent EHR systems across 4 different medical centers.

Potential biases may occur among study participants, particularly those who are technology enthusiasts or involved with clinical informatics in their health system. To help mitigate this bias, we determined a quota for each professional role at each study site to ensure representation among junior and senior providers. This mix of roles helps to include providers with varying degrees of EHR experience, provider experience, and technological astuteness.

### Future Directions

Findings from this study will be used to guide future research on information overload due to EHRs. Findings may also be used to compare information load between EHR systems, in particular EHRs that use information visualization tools compared to those that do not. Future work may include follow-up studies to assess the actual usability and impacts of visualization dashboards in real-world settings, beyond the controlled, simulated environments used in this study.

### Dissemination Plan

The results of this study will be disseminated through peer-reviewed journal publications, conference presentations, and workshops. We will also engage with health care providers, EHR vendors, and policymakers to share our findings and discuss potential implications for improving EHR design and usability. Additionally, we plan to understand the facilitators and barriers to implementing visualization dashboards in clinical settings by interviewing key stakeholders.

### Conclusions

This study aims to examine the effect of using information visualization tools on cognitive fatigue, performance, and usability among ICU providers during EHR use. Through an RCT, we will evaluate EHR usability across multiple medical centers, comparing providers’ institutional EHR systems (control) with a visualization dashboard–based EHR (intervention). Findings from this research may inform improvements in EHR interface design and usability, which may enhance provider performance, streamline care delivery, and improve patient safety outcomes.

## Abbreviations

APP: advance practice provider
EHR: electronic health record
ICU: intensive care unit
NASA-TLX: National Aeronautics and Space Administration task load index
RCT: randomized controlled trial

## Data Availability

The data collected for this study are not openly available due to reasons of sensitivity and are available from the corresponding author upon reasonable request. Data are located in controlled access data storage at the University of North Carolina at Chapel Hill.
